# Assessment of Chlorophyll-*a* Algorithms Considering Different Trophic Statuses and Optimal Bands

**DOI:** 10.3390/s17081746

**Published:** 2017-07-31

**Authors:** Salem Ibrahim Salem, Hiroto Higa, Hyungjun Kim, Hiroshi Kobayashi, Kazuo Oki, Taikan Oki

**Affiliations:** 1 Institute of Industrial Science, The University of Tokyo, 4-6-1 Komaba, Meguro-ku, Tokyo 153-8505, Japan; hjkim@iis.u-tokyo.ac.jp (H.K.); kazu@iis.u-tokyo.ac.jp (K.O.); taikan@iis.u-tokyo.ac.jp (T.O.); 2 Faculty of Engineering, Alexandria University, Lotfy El-Sied St. Off Gamal Abd El-Naser-Alexandria, Alexandria 11432, Egypt; 3 Faculty of Urban Innovation, Yokohama National University, Tokiwadai 79-5, Hodogaya, Yokohama, Kanagawa 240-8501, Japan; higa-h@ynu.ac.jp; 4 Graduate School of Interdisciplinary Research, University of Yamanashi, 4-4-37 Takeda, Kofu, Yamanashi 400-8510, Japan; kobachu@yamanashi.ac.jp

**Keywords:** bio-optical model, Red-NIR algorithm, Case 2 waters, inland lakes, Tokyo Bay, water quality

## Abstract

Numerous algorithms have been proposed to retrieve chlorophyll-*a* concentrations in Case 2 waters; however, the retrieval accuracy is far from satisfactory. In this research, seven algorithms are assessed with different band combinations of multispectral and hyperspectral bands using linear (LN), quadratic polynomial (QP) and power (PW) regression approaches, resulting in altogether 43 algorithmic combinations. These algorithms are evaluated by using simulated and measured datasets to understand the strengths and limitations of these algorithms. Two simulated datasets comprising 500,000 reflectance spectra each, both based on wide ranges of inherent optical properties (IOPs), are generated for the calibration and validation stages. Results reveal that the regression approach (i.e., LN, QP, and PW) has more influence on the simulated dataset than on the measured one. The algorithms that incorporated linear regression provide the highest retrieval accuracy for the simulated dataset. Results from simulated datasets reveal that the 3-band (3b) algorithm that incorporate 665-nm and 680-nm bands and band tuning selection approach outperformed other algorithms with root mean square error (RMSE) of 15.87 mg·m^−3^, 16.25 mg·m^−3^, and 19.05 mg·m^−3^, respectively. The spatial distribution of the best performing algorithms, for various combinations of chlorophyll-*a* (Chla) and non-algal particles (NAP) concentrations, show that the 3b_tuning_QP and 3b_680_QP outperform other algorithms in terms of minimum RMSE frequency of 33.19% and 60.52%, respectively. However, the two algorithms failed to accurately retrieve Chla for many combinations of Chla and NAP, particularly for low Chla and NAP concentrations. In addition, the spatial distribution emphasizes that no single algorithm can provide outstanding accuracy for Chla retrieval and that multi-algorithms should be included to reduce the error. Comparing the results of the measured and simulated datasets reveal that the algorithms that incorporate the 665-nm band outperform other algorithms for measured dataset (RMSE = 36.84 mg·m^−3^), while algorithms that incorporate the band tuning approach provide the highest retrieval accuracy for the simulated dataset (RMSE = 25.05 mg·m^−3^).

## 1. Introduction

Monitoring the water quality of water bodies is an essential tool for determining and controlling pollution-prone areas in order to conserve our planet. In particular, inland lakes and coastal areas should be continuously and accurately monitored. The former contains approximately 90% of the global freshwater that supports human activities [1]. The latter provides the majority of the dissolved organic carbon (DOC), which is a vital link in the global carbon cycle [2]. Water constituents’ concentrations are used as indicators to assess water quality. These constituents include chlorophyll-*a* (Chla), total suspended solids (TSS), non-algal particles (NAP), colored dissolved organic matter (CDOM) and nutrients [3,4]. Chla is an important pigment in phytoplankton that can provide clues on the trophic status, water clarity and phytoplankton biomass [5]. Indeed, in-situ and laboratory measurements are accurate for sampling points but do not provide the spatial or temporal distribution of the water constituents to monitor water quality. Remote sensing techniques, on the other hand, provide the spatial relationship for whole water bodies with repetitive coverage and historical records [6].

Water bodies can be classified into Case 1 waters (e.g., open oceans) and Case 2 waters (e.g., coastal regions and inland waters) [7]. The optical properties of Case 1 waters are dominated by phytoplankton [8,9]. The blue and green spectrum regions are used to retrieve Chla concentrations by detecting the blue absorption peak of Chla, considering the strong signal-to-noise ratio (SNR) at these wavelengths [10,11,12]. In contrast, absorption at the blue and green wavelengths in Case 2 waters depends not only on the phytoplankton, but also on NAP and CDOM [13,14]. Hence, Chla retrieval in Case 2 waters shifts from the blue and green wavelengths to the red and near-infrared (NIR) wavelengths in order to detect the red absorption peak of Chla, where the absorption is dominated by Chla [15,16]. In addition, the presence of suspended particles increases backscattering, resulting in good SNRs in the red-NIR wavelengths [17].

During the last two decades, researchers have proposed a number of algorithms to retrieve Chla from remote sensing reflectance (R_rs_) [18,19]. NASA’s ocean color (OC4E) algorithm [10], quasi-analytical algorithm (QAA) [20,21], and Garver–Siegel–Maritorena (GSM) semi-analytical algorithm [22] were proposed for Case 1 waters. Applying Case 1 algorithms in turbid waters to retrieve Chla produced significant errors, as reported by Werdell et al. [23]. The two-band ratio [13,24,25,26], three-band algorithm [15,27,28,29,30], four-band algorithm [31], normalized difference chlorophyll index [32,33], maximum chlorophyll index [34] and synthetic chlorophyll index [35] were developed for Case 2 waters. These algorithms have been applied in numerous study areas [36,37,38,39,40,41]. The optimal bands that were used for each algorithm varied among studies; for instance, Le et al. [39] used the 681-, 709-, and 754-nm MERIS central bands with the maximum chlorophyll index algorithm, while Matsushita et al. [42] employed the 665-, 709-, and 754-nm bands. Huang et al. [43] also illustrated the various bands used with the three-band algorithm at different inland waters in the USA and China.

Recently, it has been concluded that no single algorithm can provide outstanding retrieval accuracy for Chla because of the complex interactions among water constituents [44,45]. Therefore, multi-algorithms are required for various optical water properties. Moreover, a classification scheme is required to select the appropriate algorithm for each water body. Matsushita et al. [42] used the maximum chlorophyll index (MCI) to select the proper algorithm among three algorithms, namely, the ocean color V4 (OC4), two-band ratio and three-band algorithms for clear, turbid and highly turbid waters, respectively. Gower et al. [46] reported that the MCI can only detect Chla at high concentrations, which could limit the ability of MCI to be used as a classification tool. Zhang et al. [47] classified the observed data in Lake Taihu, China into three classes by K-means clustering to evaluate the performance of six algorithms. The K-means clustering is sensitive to outliers and different initial centroids of clusters can lead to different optimal solution [48,49]. The above-mentioned classifications’ attempts lack understanding the Chla retrieval strength and limitation of incorporated algorithms.

To the best of our knowledge, the aforementioned algorithms were mainly evaluated based on limited in-situ observations, which has been reported in previous research (e.g., Le et al. [39]; Zhang et al. [47]). A dataset covers wide ranges of constituents is required to evaluate these algorithms. Thus, a comparison of these algorithms with a huge dataset that covers a broad range of trophic statuses is required, particularly when the optimal bands and regression model are considered. In this study, two simulated datasets, each comprising 500,000 reflectance spectra, were used together with a measured dataset to achieve the following objectives: (1) assess the performance of seven Chla retrieval algorithms over wide ranges of Chla, NAP and CDOM concentrations; (2) evaluate the effect of different band combinations and regression approaches on the Chla retrieval accuracy; and (3) provide a reference for algorithm selection based on the concentrations of Chla and NAP, which can be used as a base for developing a classification scheme. 

## 2. Materials and Methods

### 2.1. Field and Laboratory Measurements

**Figure 1:**
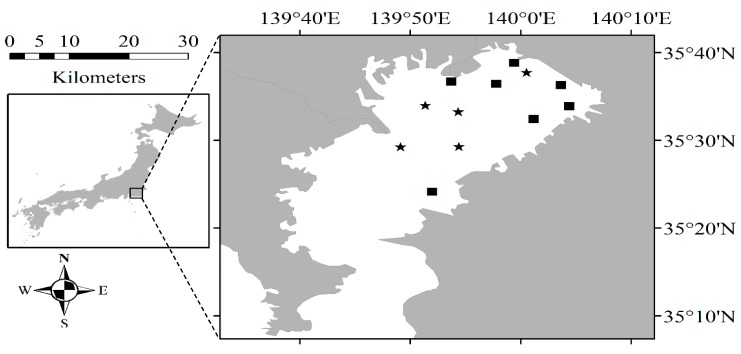
Tokyo Bay with the location of the sampling points. The squares denote stations with measurements of only water quality parameters and the stars represent stations with measurements of water surface reflectance values along with water quality parameters.

**Figure 2:**
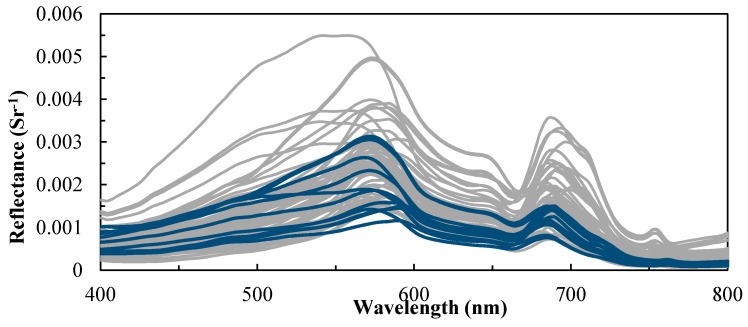
Remote sensing reflectance of 70 stations that were collected from Tokyo Bay. The gray lines denote stations with water quality parameters and remote sensing reflectance values. The blue lines represent the 12 stations with additional measurements for the inherent optical properties.

Sixteen field campaigns were conducted in Tokyo Bay (35°25′ N; 139°47′ E) from June 2010 to August 2013 (Figure 1). Tokyo Bay is located in the center of Japan. Many rivers flow into the northern and western sides of the bay. Tokyo Bay has a surface area of 1500 km^2^, with a mean and maximum water depth of 40 and 70 m, respectively. Water samples and the corresponding water surface reflectance spectra were recorded in each field campaign. Water samples were collected at a depth of 0.5 m below the surface between 09:00 and 14:00 JST. The absorption and backscattering of water constituents were also measured in four campaigns (12 sites): October 2011 (4 sites), May 2012 (3 sites), June 2012 (4 sites) and August 2012 (1 site). Remote sensing reflectance ($R_{rs}\left(\lambda \right)$) were estimated by measuring the in-water upwelling radiance $\left(L_{u}\left(0^{-},\;\lambda \right)\right)$ and downwelling irradiance $\left(E_{d}\left(0^{+},\;\lambda \right)\right)$ with three TriOS-RAMSES hyperspectral radiometers in the spectral range between 350 nm and 900 nm at a 2-nm spectral interval and a field-of-view of 7°. Irradiance sensor was attached to the ship to measure the $E_{d}\left(0^{+},\;\lambda \right)$, whereas the other two sensors were placed in the water for measuring $L_{u}\left(0^{-},\;\lambda \right)$. Both $E_{d}\left(0^{+},\;\lambda \right)$ and $L_{u}\left(0^{-},\;\lambda \right)$ are acquired for a few minutes at each station and then the irregular spectra (i.e., based on the standard deviation) are removed before averaging the remaining spectra to obtain $E_{d}\left(0^{+},\;\lambda \right)$ and $L_{u}\left(0^{-},\;\lambda \right)$ [21]. The $R_{rs}\left(\lambda \right)$ shown in Figure 2 were calculated as follows:

$$R_{rs}\left(\lambda \right)=\frac{L_{w}\left(\lambda \right)}{E_{d}\left(0^{+},\;\lambda \right)}=\left(\frac{1-\rho }{n^{2}}\right)\times \frac{L_{u}\left(0^{-},\;\lambda \right)}{E_{d}\left(0^{+},\;\lambda \right)}$$

where $L_{w}\left(\lambda \right)$ denotes the water leaving radiance, ρ refers to the Fresnel reflectance of the air sea interface with a value of 0.025 and n represents the refractive index of seawater with a value of 1.34 [50]. 

The Chla concentrations were fluorometrically measured by using a Turner Designs 10-AU fluorometer. The fluorometer was calibrated using the method described by Mitchell et al [51]. Water samples of 20 mL were filtered through 25-mm Whatman GF/F filters with a 0.7-µm pore size. The filter was immediately soaked in 6 mL of *N*,*N*-dimethylformamide (DMF) and stored in the dark at 4 °C for 4 h to extract the pigments. At each station, the Chla measurements repeated three times and the Chla concentration was obtained by averaging the three values. There are several advantages of using DMF instead of acetone including: (1) pigment extraction is less sensitive to variation of temperature; and (2) shorter time is required for pigment extraction [52]. The total suspended solids (TSS), organic suspended solids (OSS), and inorganic suspended solids (ISS) were determined gravimetrically [53]. The absorption coefficients of the non-algal particles, colored dissolved organic matter, and phytoplankton were measured with the quantitative filter technique (QFT) approach [51] by using a UV/VIS spectrophotometer (JASCO, V550, Hachioji, Japan). The vertical profiles of the total backscattering coefficients $\left(b_{b}\left(\lambda \right)\right)$ were measured at six wavelengths (420, 442, 488, 510, 550 and 676 nm) with a HydroScat-6 (HOBI Labs). The backscattering coefficients for particles $(b_{bp}\left(\lambda )\right)$ were estimated by subtracting the backscattering of pure water [36,54] from $b_{b}\left(\lambda \right)$ [55]. Table 1 summarizes the descriptive statistics of the optical water quality parameters.

### 2.2. Bio-Optical Model

Gordon et al. [11] fitted the remote sensing reflectance just beneath the water surface, $r_{rs}\;(\lambda )$, with a polynomial function of inherent optical properties (IOPs), which can be simplified as

(1) $$r_{\mathrm{rs}}\;(\lambda )=\frac{f}{Q}\times \left(\frac{b_{b}(\lambda )}{a(\lambda )+b_{b}(\lambda )}\right)$$

where a(λ) and $b_{b}(\lambda )$ are the total absorption and total backscattering coefficients, respectively; f is the light field factor; and Q denotes the light distribution factor. Kirk [56] found that f is a function of the solar altitude, which is expressed as a linear function of the cosine of the zenith angle of the refracted photons $\mu_{o}$:

(2) $$f=0.975-0.629\mu_{o}$$

The value of f/Q was 0.09 based on the Tokyo Bay in-situ measurements, which was consistent with the previously reported value from a highly turbid water body (i.e., Lake Taihu, China) [57]. Both values of a(λ) and are due to the collective contributions of pure water and optically active components within the water, which include three constituents: phytoplankton, NAP and CDOM. The total absorption a(λ) is expressed as

(3) $$a(\lambda )=a_{w}(\lambda )+a_{ph}(\lambda )+a_{NAP}(\lambda )+a_{CDOM}(\lambda )$$

where $a_{w}(\lambda )$ is the absorption coefficient of pure water [58]; and $a_{ph}(\lambda )$, $a_{NAP}(\lambda )$, and $a_{CDOM}(\lambda )$ are the absorption coefficients for phytoplankton, NAP, and CDOM, respectively (Figure S1 in Supplementary Materials). The total backscattering coefficient $b_{b}(\lambda )$ can be described as

(4) $$b_{b}(\lambda )=b_{b,w}(\lambda )+b_{b,p}(\lambda )$$

where $b_{b,w}(\lambda )$ is the backscattering coefficient of pure water [36,54] and $b_{b,p}(\lambda )$ is the backscattering coefficient of particles. The radiance just above the surface was related to the radiance just below the surface by a factor of 0.544 [59]. Hence, the remote sensing reflectance just above the water surface, $R_{\mathrm{rs}}\;(\lambda )$, can be calculated as

(5) $$R_{\mathrm{rs}}\;(\lambda )=0.544\times \frac{f}{Q}\times \left(\frac{b_{b}(\lambda )}{a(\lambda )+b_{b}(\lambda )}\right)$$

### 2.3. Simulating the Remote Sensing Reflectance

Numerous researchers have proposed many models to generate simulated R_rs_ based on bio-optical models [38,60,61]. The main difference between these models is the specific absorption and backscattering parameters [62]. The phytoplankton absorption, $a_{ph}(\lambda )$, and chlorophyll concentration, [Chla], have a proportional relationship [63] that can be expressed as

(6) $$a_{ph}(\lambda )=\left[Chla\right]\times a_{ph}^{*}(\lambda )$$

where $a_{ph}^{*}(\lambda )$ refers to the specific absorption of phytoplankton. The specific IOPs for phytoplankton, NAP, and CDOM were computed by averaging their values for the 12 stations within Tokyo Bay. The absorption spectra of both NAP and CDOM have an exponential decay distribution with increasing wavelength and can be determined from their reference values at 440 nm as follows:

(7) $$a_{NAP}(\lambda )=\left[NAP\right]\times a_{Ph}^{*}(\lambda )=\left[NAP\right]\times a_{NAP}^{*}(440)\times e^{\left(-S_{NAP}\times \left(\lambda -440\right)\right)}$$

(8) $$a_{CDOM}(\lambda )=\left[CDOM\right]\times a_{CDOM}^{*}(\lambda )=\left[CDOM\right]\times a_{CDOM}^{*}(440)\times e^{\left(-S_{CDOM}\times \left(\lambda -440\right)\right)}$$

where [NAP] is the concentration of NAP; [CDOM] refers to the CDOM absorption at 440 nm; $a_{NAP}^{*}(\lambda )$ and $a_{CDOM}^{*}(\lambda )$ are the specific absorption of NAP and CDOM at any wavelength, respectively; $a_{NAP}^{*}(440)$ and $a_{CDOM}^{*}(440)$ stand for the specific absorption of NAP and CDOM at 440 nm, respectively; and $S_{NAP}$ and $S_{CDOM}$ represent the decay slope coefficients of NAP and CDOM, respectively. The backscattering coefficient of particles, $b_{b,p}(\lambda )$, at different wavelengths was estimated by fitting the measured backscattering at the six wavelengths with an exponential function:

(9) $$b_{b,p}(\lambda )=b_{b,p}(\lambda_{o})\times {\left(\frac{\lambda }{\lambda_{o}}\right)}^{-n}$$

where n stands for the spectral slope of $b_{b,p}(\lambda )$; $b_{b,p}(\lambda_{o})$ refers to the backscattering at the reference band $\left(\lambda_{o}\right)$; and $\lambda_{o}$ was assigned to 550 nm. The segregation of $b_{b,p}(\lambda )$ into the backscattering of phytoplankton $\left(b_{b,ph}(\lambda )\right)$ and non-algal particles $\left(b_{b,NAP}(\lambda )\right)$ was based on the assumption that their contributions to $b_{b,p}(\lambda )$ were proportionally correlated to their concentrations [38]. Separating NAP from the TSS with laboratory analysis is impossible [64], so the approach of Gons et al. [65] was employed to divide the TSS into NAP and phytoplankton suspended solids (PSS). First, a linear regression model was created a relationship between Chla and OSS at 70 stations of Tokyo Bay. This relationship showed that the 1.0 mg·m^−3^ Chla concentration was relatively equivalent to 0.0687·g m^−3^ TSS. Therefore, the NAP concentration was calculated by subtracting the PSS (=0.0687 × Chla) from the TSS. 

The contribution of chlorophyll fluorescence emission to the remote sensing reflectance was simulated by Gilerson et al. [63] for Case 2 waters. The fluorescence spectral distribution follows a Gaussian shape with a peak at 685 nm, a full-width half maximum of 25 nm and a standard deviation (σ) of 10.6 nm [66]. Thus, the chlorophyll fluorescence emission reflectance $R_{rs,fl}(\lambda )$ was modeled as

(10) $$R_{rs,fl}(\lambda )=\frac{fl(685)}{1000\times E_{d}(685)}\times \exp\left[-0.5\times {\left(\frac{\lambda -685}{\sigma }\right)}^{2}\right]$$

where $E_{d}\left(685\right)$ refers to the downwelling irradiance just above the water surface at 685 nm with a value of 1.1 W·m^−2^·nm^−1^ under clear sky conditions, and fl(685) is the chlorophyll fluorescence at 685 nm, which was calculated from Gilerson et al. [67] as

(11) $$fl(685)=\frac{0.0375\left[Chla\right]}{1+0.32a_{CDOM}(440)+0.01\left[NAP\right]+0.032\left[Chla\right]}$$

The total reflectance was calculated as the sum of the reflectance that was estimated in Equations (5) and (10) [32]. Table 2 summarizes the IOPs values that used to simulate reflectance.

### 2.4. Candidate Chla Algorithms and Band Selection

During this research, seven algorithms (i.e., OC4E, two-band ratio, three-band algorithm, four-band algorithm, maximum chlorophyll index, normalized difference chlorophyll index, and synthetic chlorophyll index) were selected to assess Chla retrieval for highly turbid waters. The formulas of the seven algorithms are listed in Appendix A and a brief summary of these algorithms can be found in the Supplementary Materials (Section S1). The aforementioned algorithms are general expressions that can be applied to various band combinations of multispectral and hyperspectral data. All the possible band combinations for each algorithm were examined, resulting in 15 algorithms (Table 3). The proposed bands were the same as MERIS’s central bands. Although the MERIS mission ended in 2012, the Ocean and Land Colour Instrument (OLCI) sensor on board of the Sentinel-3 satellite was recently launched with similar configurations to MERIS and additional bands to improve atmospheric correction. The 665-nm and 680-nm bands were selected for three reasons. First, these two bands are present in most ocean color sensors (e.g., the spectral bands of the MEdium Resolution Imaging Spectrometer (MERIS) and MODerate Resolution Imaging Spectro-radiometer (MODIS) sensors); Second, these two wavelengths are interchangeable for Case 2 water algorithms; for instance, Le et al. [39] and Matsushita et al. [42] used the 680-nm and 665-nm bands with the MCI algorithms, respectively; Finally, these two bands are correlated with chlorophyll-*a*; as the 665-nm band is located near the red Chla absorption maximum in the spectral curves, while the 680-nm band is closer to the maximum fluorescence of Chla. The band tuning approach was applied to the three- and four-band algorithms to find the optimal band, as proposed by Sun et al. [69].

All 15 algorithms, except for OC4E, provided indicators that could be related to the measured constituents through the regression process. Researchers considered linear [37,70], polynomial [32,47,71,72] and power [10,73] regression approaches. Consequently, 14 out of the 15 proposed algorithms, excluding OC4E, were assessed with linear, quadratic polynomial and power regression approaches. Thus, this study proposed 43 algorithms to be evaluated. The linear, polynomial and power regressions were as follows:

(12) $$Chla=a\times a{\mathrm{lg}}_{r}+b$$

(13) $$Chla=a\times {\left(a{\mathrm{lg}}_{r}\right)}^{2}+b\times a{\mathrm{lg}}_{r}+c$$

(14) $$Chla={\left(a\times a{\mathrm{lg}}_{r}+b\right)}^{c}$$

where $Ind_{a\mathrm{lg}}$ denotes the indicators from each algorithm; and a–c are the regression coefficients. The selected power regression varies from the standard power regression expression ($Chla=a\times {\left(a{\mathrm{lg}}_{r}\right)}^{b}$) as this standard expression creates error for negative values of $a{\mathrm{lg}}_{r}$. The proposed power model was also employed by Matsushita et al. [42].

### 2.5. Accuracy Assessment of the Algorithms

The algorithms’ performance was evaluated using the root mean square error (RMSE) and the mean absolute relative error (MARE).

(15) $$RMSE=\sqrt{\frac{\sum_{i=1}^{N}{\left(X_{i,input}-X_{i,retr}\right)}^{2}}{N}}$$

(16) $$MARE=\frac{1}{N}\times \sum_{i=1}^{N}\frac{\left|\left(X_{i,input}-X_{i,retr}\right)\right|}{X_{i,input}}\times 100$$

where $X_{i,input}$ and $X_{i,retr}$ denote the input (i.e., measured or reference concentrations) and retrieved Chla concentrations, respectively; and N is the total number of samples. The log-based error (${Error}_{\log}$) was used as proposed by Zhu et al. [60] to investigate the error of the Chla concentration.

(17) $${Error}_{\log}=\log_{10}\left(X_{i,input}\right)-\log_{10}\left(X_{i,retr}\right)$$

## 3. Results and Discussion

### 3.1. Algorithm Assessment with the Simulated Reflectance

#### 3.1.1. Simulated Reflectance Assessment

**Figure 3:**
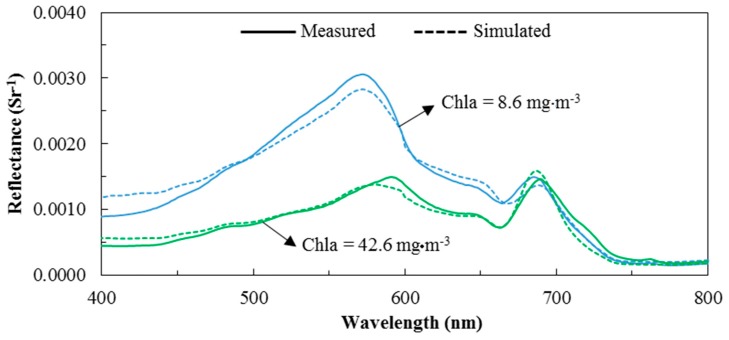
Measured versus simulated remote sensing reflectance spectra using measured inherent optical properties (IOPs) at two stations with chlorophyll-*a* (Chla) of 8.6 and 42.6 mg·m^−3^. The solid and dashed lines represent the measured and simulated reflectance, respectively.

Given the lack of extensive field data covering different tropic status, a simulated reflectance dataset is required to assess the proposed algorithms for waters that range from low to high turbidity. Bio-optical modeling is used to connect remote sensing reflectance with Chla, NAP, and CDOM concentrations. Changing any of these three constituents will change the remote sensing reflectance. Remote sensing reflectance spectra were generated based on a bio-optical model. Figure 3 illustrates a comparison between the measured and simulated reflectance spectra at two stations, whereas Figure S2 in Supplementary Materials shows the comparison of the Tokyo Bay’s twelve stations that have IOPs measurements. The simulated reflectance spectra for these two stations were generated using the bio-optical model and average value of IOPs measured in Tokyo Bay for these two stations (Table 2). This comparison revealed that the simulated reflectance spectra were similar to the measured reflectance in terms of both the spectral shape and spectral magnitude, especially in the red-NIR region, which is mainly used to substitute for Case 2 water algorithms.

**Figure 4:**
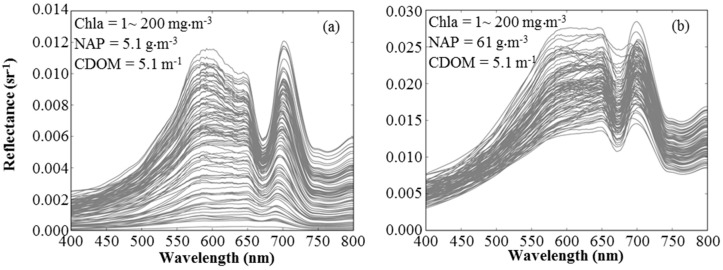
Examples of simulated reflectance spectra. Both panels show simulated reflectance spectra for chlorophyll-*a* (Chla) in ranges of 1~200 mg·m^−3^ and colored dissolved organic matter (CDOM) of 5.1 m^−1^: (**a**) reflectance spectra at low non-algal particles (NAP) (5.1 g·m^−3^); and (**b**) reflectance spectra at high NAP (61.0 g·m^−3^).

In order to generate the simulated reflectance datasets covering a wide range of constituents, the concentrations of Chla (hereafter called the reference Chla), NAP, and CDOM (the CDOM concentration refers to the absorption of CDOM at 440 nm) were changed in ranges of 1–200 (mg·m^−3^), 1–200 (g·m^−3^) and 0.1–10 (m^−1^), respectively. Increments of 2 mg·m^−3^, 2 g·m^−3^ and 0.2 m^−1^ were used for Chla, NAP and CDOM, respectively. The simulated reflectance was generated using the bio-optical model explained in Section 2.2 (Equations (3)–(11)). The values of SIOPs were randomly changed during the simulation process (Table 2, wide range). Specific phytoplankton absorption used to simulate reflectance was taken from Ciotti [74] with different weighting factors (0.1 ≤ Sf ≤ 0.5) range for microplankton and picoplankton. Two simulated datasets were generated with 500,000 reflectance spectra (i.e., 100 × 50 × 100) for each dataset. These two datasets were used for calibration and validation stages. Figure 4 demonstrates the simulated reflectance for different Chla concentrations of 1–200 mg m^−3^ and CDOM concentration of 5.1 m^−1^ for two groups of data, while the NAP was 5.1 g m^−3^ (Figure 4a) and 61 g·m^−3^ (Figure 4b). Based on these evaluations, the simulated reflectance could be a reliable dataset to assess the performance of Chla algorithms over wide ranges of Chla, NAP and CDOM concentrations.

**Figure 5:**
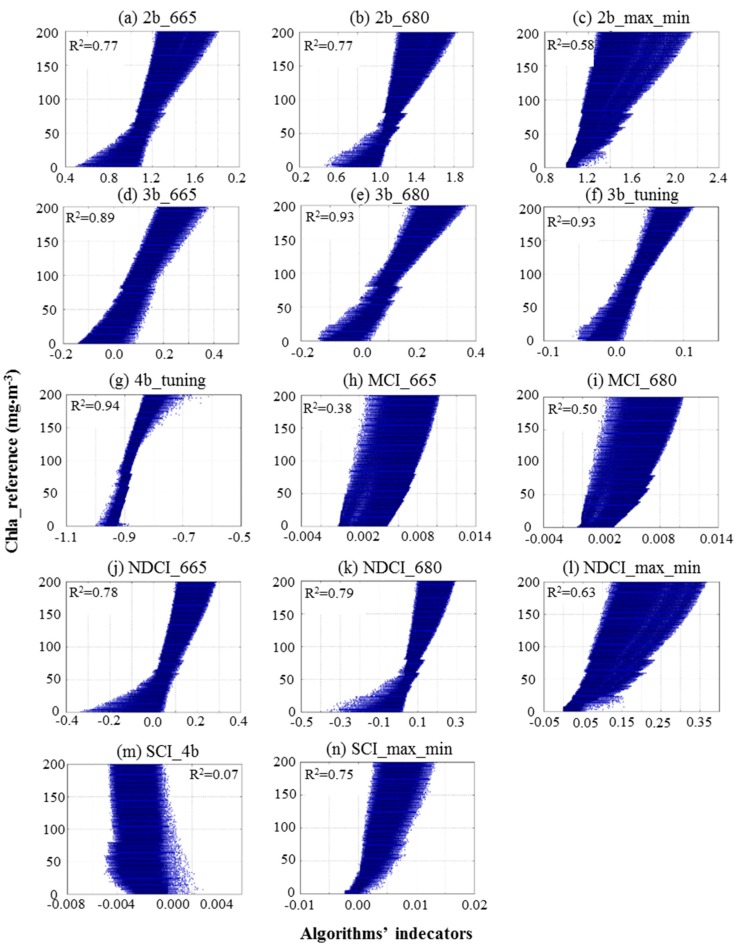
Reference reflectance versus algorithms’ ratio for the calibration dataset of simulated reflectance.

During the calibration stage, algorithms’ indicators were estimated using reflectance spectra from one of the generated simulated datasets (hereafter, calibration dataset). The algorithms’ indicators were then correlated with the reference Chla of the calibration dataset to obtain the regression coefficients of linear, quadratic polynomial and power regression approaches (Table 4). Figure 5 shows scatterplots of reference Chla versus algorithms’ indicators. The 4b_tuning, 3b_tuning, and 3b_680 revealed a relatively high correlation with coefficient of determination (R^2^) ranging from 0.93 to 0.94. The SCI_4b and OC3E algorithms introduced the lowest correlation with R^2^ < 0.07. For the validation stage, the Chla concentrations were retrieved using regression coefficients that were estimated during the calibration stage, as well as algorithms’ indicators from the second simulated dataset (hereafter, validation dataset). In the following sections, an evaluation of the retrieved Chla will be conducted.

#### 3.1.2. Overall Performance

**Figure 6:**
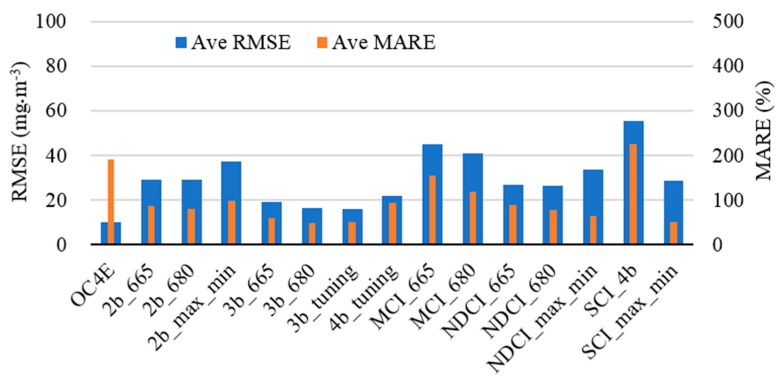
Overall assessment of the 15 algorithms in terms of root mean square error (RMSE) and mean absolute relative error (MARE) for simulated dataset. The values for RMSE and MARE were averaged for the three regression approaches of each algorithm (e.g., the RMSE of 2b_665_LN, 2b_665_QP, and 2b_665_PW were averaged as 2b_665).

The retrieval accuracy of each of the 43 algorithmic combinations was compared in terms of the R^2^, RMSE and MARE values (Table 4). Overall, the comparison of the three regression approach (i.e., linear, quadratic polynomial and power regression) for each algorithm revealed that their retrieval accuracies were comparable. The most important factor that contributed toward the algorithms’ accuracy is the algorithm indicators. Consequently, the RMSE and MARE values were averaged for the three regression approaches within each algorithm (e.g., the RMSE of 2b_665_LN, 2b_665_QP, and 2b_665_PW were averaged as 2b_665), as shown in Figure 6. The 3b_tuning (RMSE = 15.87 mg·m^−3^), 3b_680 (RMSE = 16.25 mg·m^−3^), and 3b_665 (RMSE = 19.05 mg·m^−3^) outperformed other algorithms. In contrast, the MCI_665, MCI_680, and SCI_4b algorithms showed the highest error with RMSE > 40.0 mg·m^−3^. 

The results from the SCI_4b and SCI_max_min revealed that the SCI_max_min provided less error in terms of RMSE, which decreased from 55.55 mg·m^−3^ to 28.80 mg·m^−3^ (Figure 6). These results can be attributed to the selected bands as SCI_4b did not contain the 709-nm band, which is significant to Chla retrieval; this is consistent with the findings of Gower et al. [34]. The OC4E algorithm was evaluated using relatively low Chla and NAP concentrations in ranges of 1–20 mg·m^−3^ and 1–20 g·m^−3^, respectively. Although the OC4E algorithm was originally trained using a huge dataset (2804 station) with wide ranges of Chla concentrations (0.01–64 mg·m^−3^) [75], it introduced relatively high error (RMSE = 9.89 mg·m^−3^, MARE = 191.79%). The MARE of OC4E was very high because the MARE is a relative error, which assesses the Chla retrieval over a small range of Chla values (≤20 mg·m^−3^); thus, any small difference between the reference and retrieved Chla concentrations would cause a high relative error. These results reveal the importance of executing an optimization process to determine the coefficients of OC4E with a calibration dataset to improve retrieval accuracy.

#### 3.1.3. Algorithm Performance by Considering Chla and NAP

**Figure 7:**
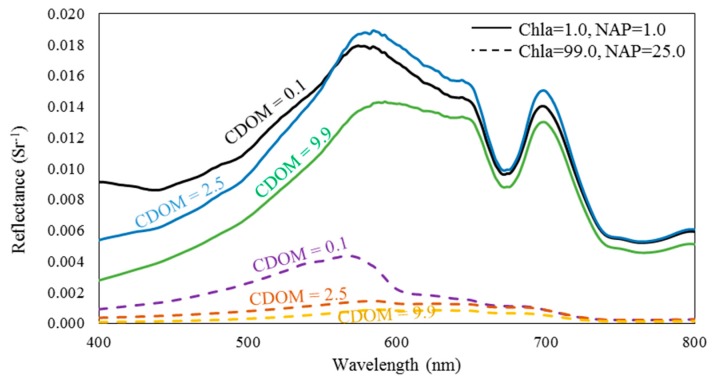
Comparing the influence of changing colored dissolved organic matter (CDOM) concentrations on the simulated reflectance spectra within the blue-green and red-NIR regions. The CDOM concentrations were 0.1, 2.5 and 9.9 m^−1^. The solid lines indicate chlorophyll-*a* (Chla) and non-algal particles (NAP) concentrations of 1.0 mg·m^−3^ and 1.0 g·m^−3^, respectively. The dashed lines indicate a Chla of 99 mg·m^−3^ and NAP of 25 g·m^−3^.

As explained, the bio-optical model links the remote sensing reflectance with IOPs (i.e., total absorption and total backscattering). The CDOM only contributes to the total absorption, while the Chla and NAP are fractions of both the absorption and backscattering. The CDOM absorption is very high in the blue wavelengths and exponentially decreases with increasing wavelength. Thus, its contribution to the total absorption in the red-NIR wavelengths is very low compared to that of phytoplankton, which has a relatively high absorption in the red-NIR wavelengths [76]. In addition, increasing the NAP increases the total backscattering and the backscattering in the NIR wavelengths. The reflectance spectra were compared for two groups of simulated reflectance to reveal the influence of changing CDOM concentrations on the simulated reflectance for blue-green and red-NIR regions (Figure 7). Within each group, the concentrations of Chla and NAP were fixed, while the CDOM was examined at three different concentrations: low (0.1 m^−1^), moderate (2.5 m^−1^) and high (9.9 m^−1^) concentrations. For both groups, the influence of changing CDOM concentrations was relatively higher in the blue-green region than in the red-NIR region. Case 2 waters’ algorithms mainly use the reflectance spectra in the red-NIR region. Accordingly, the algorithms’ retrieval accuracy was assessed for various combinations of Chla and NAP.

**Figure 8:**
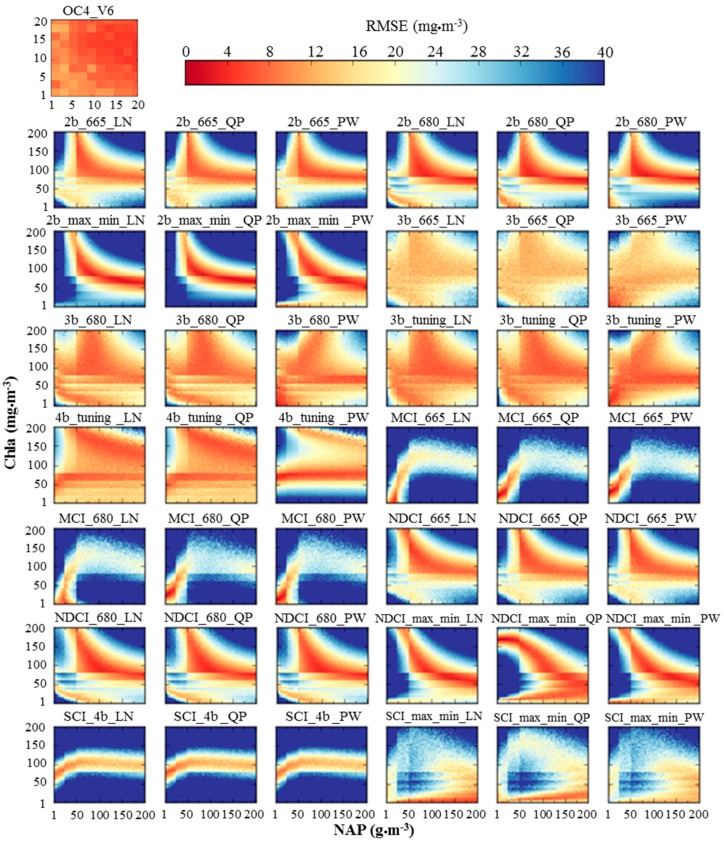
RMSEs of the 43 algorithms between the reference and retrieved chlorophyll-*a* (Chla) for each Chla and non-algal particles (NAP) combination. The Chla and NAP concentrations’ ranges of OC4E algorithm were 1–200 mg·m^−3^ and 1–200 g·m^−3^.

The RMSE of the 43 algorithms were estimated between the reference and retrieved Chla for each Chla and NAP combination (Figure 8). The red colors indicate low error, whereas the blue colors indicate high error with an upper limit of 40 mg·m^−3^ for RMSE. In general, the selection of regression approach (i.e., linear, quadratic polynomial and power regression) had low influence on the retrieval accuracy (Figure 8). For example, the 2b_665_LN, 2b_665_QP and 2b_665_PW provided similar accuracies (Figure 8). In addition, the band incorporated with each algorithm (e.g., 665-nm, 680-nm and tuning approach) had low influence on the retrieval accuracy. For instance, the 2b_665, 2b_680 and 2b_max_min with linear, quadratic polynomial and power regression approaches introduced comparable accuracy (Figure 8). However, the retrieval accuracy significantly changed across different algorithms (i.e., two-band, three-band, four-band, MCI, NDCI and SCI algorithms). 

Twelve out of the 43 combinations (3b_665, 3b_680, 3b_tuning and 4b_tuning with linear, quadratic polynomial and power regression approaches) introduced high retrieval accuracy for different concentration combinations of Chla and NAP (Figure 8). The second-tier of accurate algorithms were 2b_665, 2b_680, 2b_max_min, NDCI_665, NDCI_680, and NDCI_max_min with linear, quadratic polynomial and power models. The second-tier algorithms were accurate with moderate Chla and NAP concentrations (Figure 8). The MCI and SCI algorithms with different combinations showed the lowest retrieval accuracies. The performance of SCI_max_min was higher than SCI_4b as using the maximum and minimum technique to find the optimal band enabled SCI_max_min to be correlated with Chla concentrations. The Chla retrieval accuracy of OC4E in terms of RMSE was about 8.0 mg m^−3^ for various combinations of Chla and NAP (Figure 8). However, this retrieval accuracy can be considered of low accuracy because the OC4E was investigated in low ranges of Chla (≤20 mg·m^−3^) and NAP (≤20 g·m^−3^). The low retrieval of OC4E can be attributed to two reasons; (1) the coefficients of OC4E need to be optimized with the calibration dataset to improve the retrieval accuracy; and (2) high absorbance of NAP and CDOM along with Chla in the blue-green wavelengths could affect the accuracy of OC4E to accurately retrieve Chla.

The algorithms illustrated in Figure 8 can be classified into two groups based on the influence of NAP concentrations on the retrieval accuracy. The first group consists of twelve algorithms (i.e., 3b_665, 3b_680, 3b_tuning and 4b_tuning with linear, quadratic polynomial and power regression approaches), whereas the second group represents the rest of the algorithms. In the first group, the NAP had almost the same influence at a given Chla concentration. For example, the 3b_680_LN algorithm produced same retrieval accuracy at different NAP concentration (i.e., 1–200 g·m^−3^) and Chla concentration of 100 mg·m^−3^. In the second group, the retrieval accuracy will change for different NAP concentrations at a given Chla concentration. For instance, the retrieval accuracy of 2b_665_LN was different across different NAP concentrations (i.e., 1–200 g·m^−3^) and Chla of 150 mg·m^−3^

#### 3.1.4. The Most Accurate Algorithms among Chla and NAP Combinations.

The retrieval accuracies of the algorithms by using linear, quadratic polynomial and power regression were comparable, as concluded in Section 3.1.1. Thus, the quadratic polynomial regression approaches, along with OC4E, were compared to find the most accurate algorithms among Chla and NAP combinations, resulting in 15 algorithms listed in Table 5. A total of 10,000 combinations of Chla and NAP concentrations existed. The lowest RMSE values were selected among the 15 algorithms to find the most accurate algorithm for each combination of Chla and NAP. Table 5 summarizes the frequency of producing the minimum error for each algorithm. The 3b_tuning_QP outperformed other algorithms in terms of frequency (33.19%). In addition, the ten algorithms that required only multispectral data (i.e., OC4E and the nine multi-band algorithms, excluding all of the algorithms that required band tuning) were also examined (Table 5). The 3b_680_QP had the most outstanding accuracy, as it was the most frequent algorithm that achieved the minimum RMSE among the ten multi-band algorithms (60.52%).

**Figure 9:**
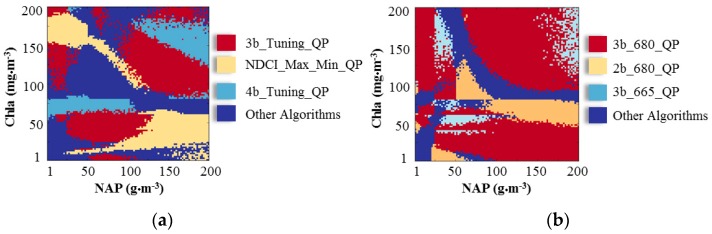
Colors illustrate the most accurate algorithms with the lowest RMSE by considering the chlorophyll-*a* (Chla) and non-algal particles (NAP) concentrations. The OC3E and quadratic polynomial algorithms were only compared (i.e., 15 algorithm combinations). (**a**) Top 3 algorithms of the 15 algorithm combinations; and (**b**) Top 3 algorithms of 10 multi-band algorithm combinations.

Figure 9 illustrates the spatial distribution of the best three algorithms by considering all 15 algorithms and the 10 multi-band algorithms. Overall, there was a complex interaction between the spatial distributions of the most accurate algorithms (Figure 9a,b). These results reveal that no single algorithm has the best accuracy for Chla retrieval and that multi-algorithms should be included to reduce the error. This finding is consistent with those of recently published results [40,44]. The four-band algorithm produced higher accuracy for high Chla and NAP concentrations, due to the fact that the four-band algorithm was proposed for highly turbid water [47].

### 3.2. Algorithm Assessment with Field Measurements

The 43 algorithms were also evaluated based on the in-situ dataset measured in Tokyo Bay (i.e., 70 samples with full water quality parameters and water surface reflectance spectra). The 70 stations were randomly divided into the following ratio: 70% as a calibration dataset and 30% for validation stage. The Chla retrieval accuracy for the 43 algorithms was assessed, as shown in Table 6. The best three algorithms in terms of R^2^, RMSE and MARE for the measured dataset were highlighted in bold (Table 6). During the calibration stage, the 3b_tuning_QP, 3b_tuning_PW and 3b_80_PW outperformed other algorithms with R^2^ ≥ 0.72. However, 2b_665_QP, 2b_665_PW, and NDCI_665_QP provided the highest retrieval accuracy with R^2^ of 0.85 and RMSE ≤ 9.87 mg·m^−3^ for the validation dataset. 

The SCI_4b algorithm with linear, quadratic polynomial, and power regression models introduced the lowest retrieval accuracy during the calibration (R^2^ ≤ 0.12) and validation stages (R^2^ ≤ 0.14, RMSE ≥ 22.80 mg·m^−3^, and MARE ≥ 297.10 g·m^−3^). In contrast, SCI_max_min_QP provided better accuracy, which can be attributed to the band selection as SCI_max_min incorporated the reflectance peak at 709 nm, while SCI_4b did not. These results reveal the importance of incorporating the 709-nm wavelength to retrieve Chla with adequate accuracy. OC4E also provided low retrieval accuracy in terms of RMSE of 21.28 mg·m^−3^. This result was observed because OC4E was proposed for applications in open oceans, where the optical properties are dominated by the Chla concentrations. In contrast, the optical properties of Tokyo Bay are influenced not only by Chla but also by NAP and CDOM, especially in the blue and green wavelengths, which are used to substitute for the OC4E algorithm. Therefore, OC4E failed to correlate the measured reflectance with the Chla concentrations. 

**Figure 10:**
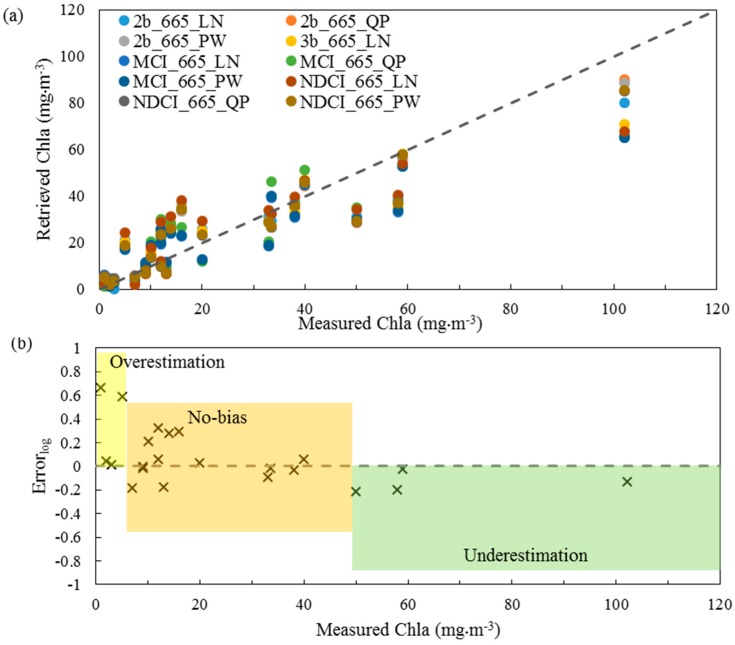
Assessment of the ten best algorithms of the in-situ dataset: (**a**) measured versus retrieved chlorophyll-*a* (Chla); and (**b**) errors versus the measured Chla, where the errors were estimated between the measured and mean retrieved Chla of the ten algorithms.

The Chla retrieval accuracies of the ten algorithms with the lowest RMSE of in-situ dataset were evaluated by comparing the measured and retrieved Chla (Figure 10a) and by calculating Error_log_ between the measured and retrieved Chla (Figure 10b). A common trend existed among these algorithms, which overestimated low Chla concentrations (Chla ≤ 6.0 mg·m^−3^) with Error_log_ > 0.0 and underestimated high Chla concentrations (Chla > 50.0 mg·m^−3^) with Error_log_ < 0.0, as shown in Figure 10a,b. No bias occurred for Chla concentrations from 7.0 to 50 mg·m^−3^. The results revealed the limitation of the investigated algorithms to accurately retrieve low and high Chla concentrations, which implies the importance of developing new algorithms that can reduce the influence of NAP and CDOM to accurately retrieve the Chla concentrations in turbid water.

### 3.3. Validity of the Simulated Reflectance Dataset’s Models on Chla Retrieval in Tokyo Bay

Although the current study was not intended to provide regression relationships from the simulated dataset to retrieve Chla concentrations in local water bodies, the retrieval accuracy of simulated dataset’s models (i.e., regression models summarizes in Table 4 obtained during calibration stage) was investigated using Tokyo Bay dataset. The measured Chla concentrations were compared with the retrieved Chla using the relationships of the 43 algorithms summarized in Table 4. In general, most of the algorithms overestimated Chla concentrations and many algorithms produced negative Chla values, especially with linear regression (Figure S3 in Supplementary Materials). Although the three-band and four-band algorithms were the most accurate algorithms in terms of R^2^ > 0.56 (i.e., 3b_tuning_LN, 3b_tuning_QP, 4b_tuning_LN and 4b_tuning_QP) (Table S1 in Supplementary Materials), they produced negative Chla concentration, particularly for low measured Chla (Figure S3f,g in Supplementary Materials). The 3b_680_LN and 4b_tuning_PW had the lowest RMSE of 17.56 and 17.14 mg·m^−3^, respectively. There was good agreement between the measured and retrieved Chla from 3b_680_LN and 4b_tuning_PW algorithms without negative values (Figure S3e,g in Supplementary Materials). The lowest algorithm in terms of MARE was 3b_665_PW (86.08%) (Table S1 in Supplementary Materials), which overestimated low Chla concentrations. Figure S3h,i in Supplementary Materials reveals the limitation of the MCI by producing same retrieved Chla for different measured Chla. These results can be attributed to the poor correlation between reference Chla and the algorithms’ indicators (Figure 5h,i). Similarly, the SCI_4b provided the lowest retrieval accuracy. 

### 3.4. Comparing the Algorithms’ Performance for Measured and Simulated Datasets

**Figure 11:**
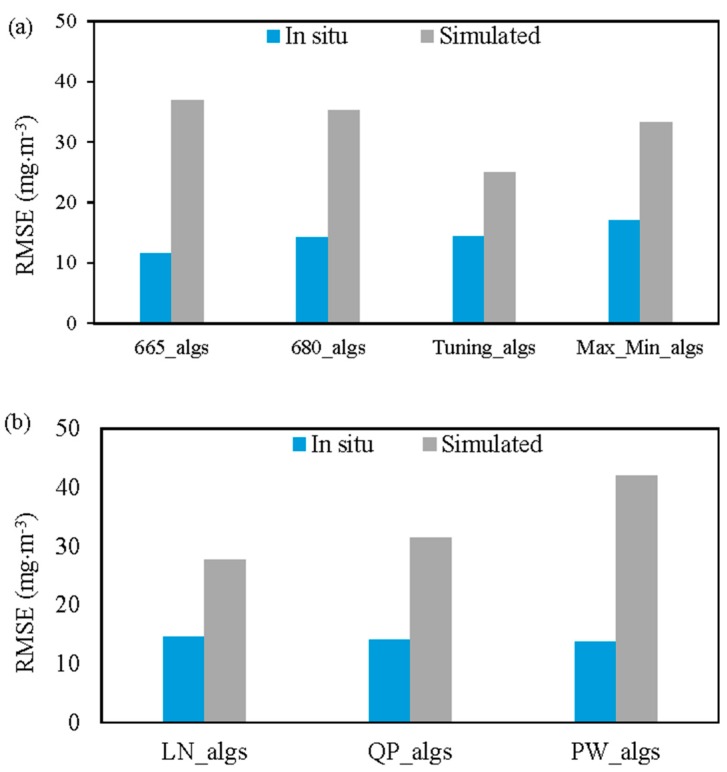
Comparison of the average retrieval accuracy of the in-situ and simulated datasets in terms of RMSE: (**a**) 665_algs, 680_algs, tuning_algs, and Max_Min_algs denote the average retrieval for all the algorithms that included the 665-nm band, 680-nm band, band tuning and maximum and minimum, respectively; and (**b**) LN_algs, QP_algs, and PW_algs represent the average of all the algorithms with linear, quadratic polynomial, and power regression models, respectively.

The overall retrieval accuracy of the band selection (e.g., algorithms with the 665-nm or band tuning) and regression models (i.e., linear, quadratic polynomial, and power models) were compared for the measured (i.e., the 70 samples), and simulated datasets (Figure 11). Except for OC4E and SCI_4b, which provided the lowest retrieval accuracy, the other 39 of the 43 algorithms were classified into four groups: algorithms with the 665-nm band (665_algs), algorithms with the 680-nm band (680_algs), algorithms that incorporated band tuning (Tuning_algs), and algorithms that utilized the maximum and minimum approach (Max_min_algs). The retrieval accuracy in terms of the RMSE for all of the algorithms within each group was averaged (Figure 11a). The Tuning_algs outperformed the other three groups for simulated dataset with RMSE of 25.05 mg·m^−3^, while the 665_algs provided the highest retrieval accuracy with RMSE of 11.58 mg. The second most accurate group varied among the two datasets: Max_Min_algs and 680_algs were the second most accurate for the simulated (RMSE = 33.21 mg·m^−3^) and in-situ datasets (RMSE = 14.23 mg·m^−3^), respectively. The 665_algs algorithm had the lowest retrieval accuracy for the simulated dataset with RMSE of 36.84 mg·m^−3^. The Max_Min_algs had the worst performance for the in-situ dataset with RMSE of 17.09 mg·m^−3^. 

Similarly, 39 of the 43 algorithms, excluding the OC4E and SCI_4b algorithms, were classified into three groups: namely, LN_algs, QP_algs, and PW_algs, which were estimated by averaging the Chla retrieval accuracy for all the algorithms with the linear, quadratic polynomial and power regression models, respectively (Figure 11b). In general, the regression approach (i.e., LN, QP, and PW) had more influence on the simulated dataset than the measured dataset due to the fact that the variation of the remote sensing reflectance among the simulated dataset was very large, as shown in Figure 4a, comparing with measured reflectance (Figure 2). The LN_algs had the best retrieval accuracy for the simulated dataset (RMSE = 27.71 mg·m^−3^), whereas the QP_algs had the lowest retrieval accuracy for simulated dataset in terms of RMSE of 42.03 mg·m^−3^. The accuracy for in-situ measurements ranged between 13.77 mg·m^−3^ and 14.55 mg·m^−3^. 

## 4. Conclusions

In this study, the performances of seven Chla algorithms were investigated with all possible band combinations and three regression models (i.e., linear, quadratic polynomial and power regression approaches). In total, 43 algorithms were assessed based on in-situ and simulated datasets. Two simulated datasets that covered wide ranges of Chla (1–200 mg·m^−3^), NAP (1–200 g·m^−3^), and CDOM (0.1–10 m^−1^) concentrations were generated to calibrate and validate the proposed algorithms. Each of the simulated dataset comprised 500,000 reflectance spectra. Having a large pool of simulated reflectance enabled us to thoroughly evaluate the Chla algorithms. Across all algorithms, the 2b_665_QP and 4b_tuning_LN algorithms outperformed the other algorithms for measured (R^2^ = 0.85%, RMSE = 9.64 mg·m^−3^) and simulated datasets (R^2^ = 0.93%, RMSE = 14.73 mg·m^−3^). The SCI algorithms showed the highest error for both datasets, with average RMSE of 22.95 mg·m^−3^ and 55.55 mg·m^−3^ for measured and simulated datasets, respectively. The spatial distribution of the most accurate algorithms among 15 algorithms (i.e., OC4E and all of the algorithms incorporated the quadratic polynomial regression approach) for the 10,000 combinations of Chla, and NAP revealed that the three-band incorporated tuning selection approach outperformed other algorithms with minimum RMSE frequency of 33.19%. In addition, the spatial distribution highlighted the importance of incorporating multi-algorithms to improve retrieval accuracy. The two-, three-, and four-band, and NDCI algorithms tend to have acceptable accuracy among measured and simulated datasets, while some other algorithms have high error (i.e., SCI algorithms). Overall, the regression approach has more influence on simulated datasets than the measured dataset, due to the wide range of simulated datasets magnifying the influence of the fitting process. In addition, the power regression can cause errors for negative values of the algorithm’s ratio. As a result, the linear and quadratic polynomial regression is recommended for simulated and measured datasets, respectively. 

## Figures and Tables

**Table 1:** Descriptive statistics of the water quality parameters that were measured in Tokyo Bay.

	Min	Max	Mean	Median	Stdv	CV (%)
All samples (*n* = 70)						
Chla (mg·m^−3^)	2.90	97.32	24.86	16.50	21.76	87.53
TSS (g·m^−3^)	2.99	26.29	8.04	7.30	3.91	48.63
ISS (g·m^−3^)	0.50	17.20	3.78	3.16	2.72	71.81
OSS (g·m^−3^)	0.32	12.28	4.36	4.18	2.34	53.74
Samples with IOPs (*n* = 12)						
Chla (mg·m^−3^)	2.90	42.60	18.31	11.85	14.99	81.87
TSS (g·m^−3^)	2.99	9.86	6.34	6.81	2.24	35.38
ISS (g·m^−3^)	0.81	6.13	2.96	2.22	1.63	55.24
OSS (g·m^−3^)	0.96	7.16	3.39	2.90	1.75	51.63
a_ph_ (440) (m^−1^)	0.23	1.03	0.65	0.60	0.27	41.30
a_NAP_ (440) (m^−1^)	0.14	0.34	0.22	0.21	0.07	31.75
a_CDOM_ (440) (m^−1^)	0.06	0.47	0.25	0.19	0.14	56.23
b_b,p_ (442) (m^−1^)	0.01	0.04	0.02	0.03	0.01	41.16

a_ph_ (440), a_NAP_ (440) and a_CDOM_ (440) represent the measured absorption coefficients for phytoplankton, NAP and CDOM at 440 nm, respectively; b_b,p_ (442) denotes the measured backscattering at 442 nm; *n* represents the number of samples; Stdv stands for standard deviation; and CV denotes coefficient of variation = (Stdv/Mean) × 100.

**Table 2:** Values of Specific inherent optical properties (SIOPs) used in the bio-optical model to generate simulated reflectance.

SIOPs	Tokyo Bay *	Wide Range **
a*_NAP_ (440) m^2^·g^−1^	0.03483	0.02–0.1
a*_CDOM_ (440)	1.0	1.0
b*_b,ph_ (550)	0.000204	0.0001–0.002
b*_b,NAP_ (550)	0.00296	0.001–0.02
S_NAP_	0.00899	0.007–0.015
S_CDOM_	0.01547	0.01–0.02
*n*	1.25848	0.5–2.2

* Based on Tokyo Bay in-situ measurements; ** based on the IOCCG database (2006) [68].

**Table 3:** Investigated algorithms with the proposed bands for each algorithm combination.

No.	Investigated Algorithms	Algorithms’ Abbreviation	Proposed Bands				References
			*λ*_1_	*λ*_2_	*λ*_3_	*λ*_4_	
1	Ocean Color 4	OC4E	443	490	510	560	O’Reilly et al. [10]
2	Two-band ratio	2b_665	665	709			Gons [13]
3		2b_680	680	709			
4		2b_max_min	Min (660−690)	Max (690–710)			
5	Three-band algorithm	3b_665	665	709	754		Dall’Olmo et al. [15]
6		3b_680	680	709	754		
7		3b_tuning	Band tuning				
8	Four-band algorithm	4b_tuning	Band tuning				Le et al. [31]
9	Maximum chlorophyll index	MCI_665	665	709	754		Gower et al. [34]
10		MCI_680	680	709	754		
11	Normalized difference chlorophyll index	NDCI_665	665	709			Mishra et al. [32]
12		NDCI_680	680	709			
13		NDCI_max_min	Min (660–690)	Max (690–710)			
14	Synthetic chlorophyll index	SCI_4b	560	620	665	681	Shen et al. [35]
15		SCI_max_min	Max (540–590)	Max (630–650)	Min (660–690)	Max (690–710)	

**Table 4:** Algorithms’ evaluation during the calibrations and validation stages of simulated dataset.

Algorithms	Calibration				Validation		
	R^2^	a	b	c	R^2^	RMSE	MARE
OC4E	0.05	---	---	---	---	**9.89**	191.79
2b_665_LN	0.77	304.32	−261.24	---	0.77	27.50	81.86
2b_665_QP	0.75	110.24	30.51	−94.57	0.75	29.08	81.23
2b_665_PW	0.72	16.46	−8.21	1.88	---	30.66	98.62
2b_680_LN	0.78	295.80	−254.96	---	0.78	27.33	70.58
2b_680_QP	0.75	104.92	29.94	−90.03	0.75	29.15	73.82
2b_680_PW	0.72	14.63	−6.79	1.92	0.72	30.85	96.34
2b_max_min_LN	0.58	213.29	−182.64	---	0.58	37.39	104.33
2b_max_min_QP	0.53	62.12	25.42	−45.29	0.53	39.44	126.63
2b_max_min_PW	0.63	2002.34	−2002.34	0.72	0.63	34.98	61.85
3b_665_LN	0.89	626.91	31.19	---	0.90	18.71	59.50
3b_665_QP	0.89	30.27	620.36	31.31	0.90	18.71	58.93
3b_665_PW	0.88	66.33	9.32	1.62	---	19.80	57.97
3b_680_LN	**0.93**	624.45	27.70	---	**0.93**	15.62	46.11
3b_680_QP	**0.93**	−109.79	650.00	27.09	**0.93**	15.58	48.82
3b_680_PW	0.91	63.28	8.92	1.63	---	17.55	48.40
3b_tuning_LN	**0.93**	1931.03	34.98	---	**0.93**	15.09	**45.31**
3b_tuning_QP	**0.93**	−1121.35	2007.35	34.61	**0.93**	**15.05**	46.90
3b_tuning_PW	0.91	137.36	7.98	1.79	---	17.46	58.79
4b_tuning_LN	**0.94**	1315.75	1252.01	---	**0.93**	**14.73**	**40.10**
4b_tuning_QP	**0.94**	41.99	−37.02	32.41	**0.94**	25.61	118.63
4b_tuning_PW	0.82	−0.03	0.96	−291.39	0.81	25.61	118.63
MCI_665_LN	0.38	19,068.81	−9.17	---	0.38	45.45	154.44
MCI_665_QP	0.40	1,208,885.88	6474.20	19.05	0.40	44.83	152.70
MCI_665_PW	0.40	62.74	1.63	6.63	0.40	44.77	155.56
MCI_680_LN	0.50	20,525.57	−8.56	---	0.50	40.88	115.11
MCI_680_QP	0.50	1,296,821.88	7055.46	21.35	0.50	40.76	123.71
MCI_680_PW	0.50	3201.62	4.90	1.48	0.50	40.67	116.69
NDCI_665_LN	0.78	724.11	41.93	---	0.78	27.18	93.75
NDCI_665_QP	0.78	462.70	661.01	41.73	0.78	26.90	83.78
NDCI_665_PW	0.71	3.88	1.30	3.61	---	---	---
NDCI_680_LN	0.79	720.36	38.49	---	0.79	26.56	80.09
NDCI_680_QP	0.79	229.99	684.93	38.68	0.79	26.49	75.50
NDCI_680_PW	0.77	3.53	1.29	2.53	---	---	---
NDCI_max_min_LN	0.63	635.72	15.15	---	0.63	34.98	76.17
NDCI_max_min_QP	0.70	−2220.14	1309.20	−23.60	0.70	31.71	60.07
NDCI_max_min_PW	0.65	1819.05	0.22	0.84	0.65	34.02	57.33
SCI_4b_LN	0.07	−16,818.44	61.55	---	0.07	55.53	223.13
SCI_4b_QP	0.07	798,889.88	−12,357.51	66.96	0.07	55.59	226.13
SCI_4b_PW	0.08	−39,036.16	112.38	0.87	---	55.52	222.66
SCI_max_min_LN	0.75	18,622.68	21.93	---	0.75	28.85	**41.88**
SCI_max_min_QP	0.77	−824,219.81	25,764.77	12.42	0.77	27.98	52.44
SCI_max_min_PW	0.74	6639.74	15.67	1.22	0.74	29.58	56.79

The highest three performing algorithms were highlighted in bold. The lowest three performing algorithms were single-underlined. LN, QP, and PW stand for linear, quadratic polynomial, and power regression approaches, respectively.

**Table 5:** Frequency of each algorithm to achieve the minimum RMSE of the 10,000 combinations of Chla and NAP concentrations. The evaluation executed among OC4E and quadratic polynomial algorithms only.

	Frequency	
Algorithms	All 15 Algorithms (%)	Multi-Band Algorithms Only (%)
OC4E	0.63	0.76
2b_665_QP	0.49	2.12
2b_680_QP	3.55	**12.08**
2b_max_min_QP	5.35	---
3b_665_QP	1.45	**8.8**
3b_680_QP	11.43	**60.52**
3b_tuning_QP	**33.19**	---
4b_tuning_QP	**13.04**	---
MCI_665_QP	0.87	1.64
MCI_680_QP	0.92	1.16
NDCI_665_QP	1.54	4.03
NDCI_680_QP	4.58	7.24
NDCI_max_min_QP	**18.75**	---
SCI_4b_QP	1.02	1.65
SCI_max_min_QP	3.19	---

The best three algorithms in terms of frequency were highlighted in bold.

**Table 6:** Algorithms’ evaluation during the calibrations and validation stages of in-situ dataset.

	Calibration				Validation		
Algorithms	R^2^	a	b	c	R^2^	RMSE	MARE
OC4E	0.47	---	---	---	---	21.28	**51.55**
2b_665_LN	0.61	64.78	−39.70	---	0.82	10.65	75.14
2b_665_QP	0.62	22.91	13.78	−13.88	**0.85**	**9.64**	63.38
2b_665_PW	0.62	19.01	−9.35	1.38	**0.85**	**9.70**	61.91
2b_680_LN	0.61	133.70	−66.84	---	0.65	14.50	**53.62**
2b_680_QP	0.64	171.95	−120.72	22.83	0.70	13.98	77.76
2b_680_PW	0.65	31.51	−14.01	1.50	0.69	13.97	54.99
2b_max_min_LN	0.35	39.58	−38.36	---	0.58	16.23	100.85
2b_max_min_QP	0.38	−28.68	138.09	-118.21	0.46	17.97	127.73
2b_max_min_PW	0.35	56.40	−56.40	0.92	0.57	16.37	106.55
3b_665_LN	0.54	215.91	24.43	---	0.77	12.11	84.98
3b_665_QP	0.55	−341.33	246.28	27.00	0.69	13.62	103.70
3b_665_PW	0.54	218.32	24.71	1.00	---	12.88	75.31
3b_680_LN	0.70	547.28	71.46	---	0.60	15.96	112.84
3b_680_QP	0.71	1524.13	749.73	75.25	0.66	14.18	81.92
3b_680_PW	**0.72**	137.32	20.28	1.44	---	14.40	66.97
3b_tuning_LN	0.72	580.64	68.08	---	0.61	15.51	124.89
3b_tuning_QP	**0.73**	1923.03	804.47	71.34	0.69	13.79	107.45
3b_tuning_PW	**0.73**	122.83	16.73	1.52	---	13.68	97.76
4b_tuning_LN	0.70	334.81	59.03	---	0.60	15.71	120.98
4b_tuning_QP	0.71	452.07	399.09	58.98	0.66	14.20	106.46
4b_tuning_PW	0.71	79.25	16.46	1.46	---	13.73	91.86
MCI_665_LN	0.63	53,369.70	3.50	---	0.80	12.45	70.25
MCI_665_QP	0.67	−30,829,280.00	95,144.16	−3.75	0.73	12.80	78.27
MCI_665_PW	0.64	78,770.46	2.41	0.92	0.79	12.49	67.51
MCI_680_LN	0.63	83,225.49	20.74	---	0.75	13.63	106.53
MCI_680_QP	0.65	−43,715,440.00	104,539.34	22.42	0.71	13.57	93.69
MCI_680_PW	0.63	103,293.36	24.58	0.95	0.74	13.65	107.54
NDCI_665_LN	0.58	132.06	28.17	---	0.73	12.87	93.67
NDCI_665_QP	0.62	254.63	134.28	22.35	**0.85**	**9.87**	70.40
NDCI_665_PW	0.62	7.19	3.28	2.63	0.85	9.87	60.69
NDCI_680_LN	0.57	190.57	62.13	---	0.63	15.01	61.02
NDCI_680_QP	0.66	628.31	415.40	75.40	0.69	13.93	83.35
NDCI_680_PW	0.65	25.16	9.68	1.89	0.68	14.05	**52.37**
NDCI_max_min_LN	0.37	140.87	−5.21	---	0.52	16.91	118.44
NDCI_max_min_QP	0.37	16.69	133.54	−4.62	0.53	16.84	116.43
NDCI_max_min_PW	0.37	54.45	0.01	1.29	0.55	16.51	100.26
SCI_4b_LN	0.11	25,685.39	1.36	---	0.14	22.80	297.10
SCI_4b_QP	0.12	−9,940,877.00	47,537.92	−9.19	0.11	23.07	311.24
SCI_4b_PW	0.12	178,020.55	−44.51	0.68	0.12	22.99	305.93
SCI_max_min_LN	0.52	36,992.36	−20.91	---	0.51	17.62	131.03
SCI_max_min_QP	0.52	−833,195.81	39,538.18	−22.57	0.50	17.60	130.40
SCI_max_min_PW	0.52	12,071.70	−3.96	1.32	0.51	17.77	136.53

The highest three performing algorithms were highlighted in bold. The lowest three performing algorithms were single-underlined. Linear regression (LN) as expressed in Equation (12) (Chla = a × ind_alg_ + b) LN, QP, and PW stand for linear, quadratic polynomial, and power regression approaches.

## Supplementary Materials

The following are available online at http://www.mdpi.com/1424-8220/17/8/1746/s1.

## Appendix A

The formulas of the seven Chla retrieval algorithms are:

OC4E

$$Chla=10^{\left(0.3255-2.7677x+2.4409x^{2}-1.1288x^{3}-0.499x^{4}\right)}$$

$$x=\log_{10}\left(R_{rs}^{1}/R_{rs}^{2}\right)$$

$$R_{rs}^{1}/R_{rs}^{2}=\max\left(\frac{Rrs(\lambda_{1})}{Rrs(\lambda_{4})},\frac{Rrs(\lambda_{2})}{Rrs(\lambda_{4})},\frac{Rrs(\lambda_{3})}{Rrs(\lambda_{4})}\right)$$

Two-band ratio

$$Chla\propto R_{rs}(\lambda_{2})/R_{rs}(\lambda_{1})$$

Three-band algorithm

$$Chla\propto \left(R_{rs}^{-1}(\lambda_{1})-R_{rs}^{-1}(\lambda_{2})\right)\times R_{rs}(\lambda_{3})$$

Four-band algorithm

$$Chla\propto \left(R_{rs}^{-1}(\lambda_{1})-R_{rs}^{-1}(\lambda_{2})\right)/\left(R_{rs}^{-1}(\lambda_{4})-R_{rs}^{-1}(\lambda_{3})\right)$$

Maximum chlorophyll index (MCI)

$$MCI=R_{rs}(\lambda_{2})-R_{rs}(\lambda_{1})\times \left[\frac{\lambda_{2}-\lambda_{1}}{\lambda_{3}-\lambda_{1}}\times R_{rs}(\lambda_{3})-R_{rs}(\lambda_{1})\right]$$

Normalized difference chlorophyll index (NDCI)

$$Chla\propto \left(R_{rs}(\lambda_{2})-R_{rs}(\lambda_{1})\right)/\left(R_{rs}(\lambda_{2})+R_{rs}(\lambda_{1})\right)$$

Synthetic chlorophyll index (SCI)

$$SCI=H_{chl}-H_{\Delta }$$

$$H_{chl}=\left[R_{rs}(\lambda_{4})+\frac{\lambda_{4}-\lambda_{3}}{\lambda_{4}-\lambda_{2}}\times \left(R_{rs}(\lambda_{2})-R_{rs}(\lambda_{4})\right)\right]-R_{rs}(\lambda_{3})$$

$$H_{\Delta }=R_{rs}(\lambda_{2})-\left[R_{rs}(\lambda_{4})-\frac{\lambda_{4}-\lambda_{2}}{\lambda_{4}-\lambda_{1}}\times \left(R_{rs}(\lambda_{1})-R_{rs}(\lambda_{4})\right)\right]$$
