# Supplementary material for: Assessment of Chlorophyll-a Algorithms Considering Different Trophic Statuses and Optimal Bands
Source: Sensors (Basel). 2017 Jul 31;17(8):1746. doi: 10.3390/s17081746 (PMC5579528; doi:10.3390/s17081746)
Supplement: Supplementary file 1 [file sensors-17-01746-s001.pdf]

Assessment of chlorophyll-a algorithms considering different trophic statuses and optimal bands

Supplementary Material

Salem Ibrahim Salem[1,*], Hiroto Higa[2], Hyungjun Kim[1], Hiroshi Kobayashi[3], Kazuo Oki[1], and Taikan Oki[1]

1. Candidate chlorophyll-a algorithms

Various algorithms were introduced based upon the bio-optical model with various assumptions. Gons [1] outlined the first two-band ratio to retrieve the chlorophyll-a (Chla) concentration, which replaced the blue and green wavelengths with red and NIR wavelengths:

$$Chla \propto R_{rs}(\lambda_2) / R_{rs}(\lambda_1) \approx \left(a_{ph}(\lambda_1) + a_W(\lambda_1)\right) / a_w(\lambda_2) \quad (1)$$

where λ_1 and λ_2 are the red and NIR wavelengths, respectively. Three assumptions govern this algorithm: 1) the absorption at (λ_1) is dominated by phytoplankton; 2) the absorption at (λ_2) is dominated by water; and 3) the backscattering is independent of λ between λ_1 and λ_2. The normalized difference chlorophyll index (NDCI) is a modified version of the two-band ratio [2]. The NDCI was proposed to reduce any uncertainties from seasonal solar azimuth differences and atmospheric contributions at those wavelengths by taking the difference in the numerator and the sum in the denominator for $R_{rs}(\lambda_1)$ and $R_{rs}(\lambda_2)$. The NDCI was originally developed to retrieve the Chla concentration from MERIS products and can be expressed as follows:

$$Chla \propto \left(R_{rs}(\lambda_2) - R_{rs}(\lambda_1)\right) / \left(R_{rs}(\lambda_2) + R_{rs}(\lambda_1)\right) \quad (2)$$

Both the two-band ratio and NDCI neglect the absorption of NAP and CDOM, which is no longer valid with increasing turbidity, where the absorption of both NAP and CDOM are significant. Therefore, the three-band algorithm was introduced [3]:

$$Chla \propto \left(R_{rs}^{-1}(\lambda_1) - R_{rs}^{-1}(\lambda_2)\right) * R_{rs}(\lambda_3) \approx \left(a_{ph}(\lambda_1) + a_W(\lambda_1) - a_w(\lambda_2)\right) / a_w(\lambda_3) \quad (3)$$

where λ_1 is the red wavelength, and λ_2 and λ_3 are the NIR wavelengths. The assumptions of the three-band algorithm are 1) the Chla absorption at (λ_1) >> that at (λ_2); 2) the absorption at (λ_3) is dominated by water; 3) the backscattering is independent of λ between λ_1 and λ_2; and 4) the absorption of NAP and CDOM at λ_1 and λ_2 is close and the difference between λ_1 and λ_2 eliminates their effect. In highly turbid water, the previous assumptions of the three-band algorithm become invalid because of increasing TSS concentration, which causes significant absorption and backscattering in the NIR region [4]. A four-band algorithm was

proposed by Le et al. [5] to improve the three-band algorithm in highly turbid water by considering the absorption and backscattering of suspended solids and pure water. $R_{rs}(\lambda_3)$ was replaced by ($R_{rs}^{-1}(\lambda_4) - R_{rs}^{-1}(\lambda_3)$) to reduce the absorption of pure water, as well as the absorption and backscattering of suspended solids. The four-band algorithm is expressed as

$$Chla \propto \left(R_{rs}^{-1}(\lambda_1) - R_{rs}^{-1}(\lambda_2)\right) / \left(R_{rs}^{-1}(\lambda_4) - R_{rs}^{-1}(\lambda_3)\right) \approx \left(a_{ph}(\lambda_1) + a_W(\lambda_1) - a_w(\lambda_2)\right) / \left(a_w(\lambda_4) - a_w(\lambda_3)\right) \quad (4)$$

Gower et al. [6] adopted the maximum chlorophyll index (MCI) for MERIS sensor to detect the maximum reflectance at 709 nm from baseline wavelengths of 685 nm and 754 nm. To distinguish the water pixels of MERIS level 1 products from others (i.e., land, cloud and sun glint pixels), the MCI applied for pixels that the radiance values at 865 nm were less than 15 mW m^{-2} sr^{-1} nm^{-1} [7]. The MCI is as follows:

$$MCI = R_{rs}(\lambda_2) - R_{rs}(\lambda_1)\left[\frac{\lambda_2 - \lambda_1}{\lambda_3 - \lambda_1} R_{rs}(\lambda_3) - R_{rs}(\lambda_1)\right] \quad (5)$$

where λ_1 , λ_2 and λ_3 refer to the 681-, 709- and 754-nm wavelengths. The synthetic chlorophyll index (SCI) was also developed for MERIS sensor to detect the reflectance trough (H_{chl}) at 665 nm from baseline wavelengths at 620 nm and 681 nm because of the maximum absorption of phytoplankton [8]. A correction factor (H_Δ) was introduced to eliminate backscattering from high suspended solids. The absorption of CDOM for wavelengths larger than 555 nm was assumed to be negligible, and the SCI is expressed as

$$H_{chl} = \left[R_{rs}(\lambda_4) + \frac{\lambda_4 - \lambda_3}{\lambda_4 - \lambda_2}\left(R_{rs}(\lambda_2) - R_{rs}(\lambda_4)\right)\right] - R_{rs}(\lambda_3) \quad (6)$$

$$H_\Delta = R_{rs}(\lambda_2) - \left[R_{rs}(\lambda_4) - \frac{\lambda_4 - \lambda_2}{\lambda_4 - \lambda_1}\left(R_{rs}(\lambda_1) - R_{rs}(\lambda_4)\right)\right] \quad (7)$$

$$SCI = H_{chl} - H_\Delta \quad (8)$$

where λ_1 , λ_2 , λ_3 and λ_4 are the wavelengths 560, 620, 665 and 681 nm, respectively. While all the aforementioned algorithms were developed for Case 2 waters, the ocean color V4 (OC4) [9] algorithm, which was established for Case 1 waters and MERIS sensor, was also assessed for Chla concentrations less than 20 mg m^{-3}. OC4 is expressed as

$$Chla = 10^{\left(0.3255 - 2.7677x + 2.4409x^2 - 1.1288x^3 - 0.499x^4\right)} \quad (9)$$

$$x = \log_{10}\left(R_{rs}^1 / R_{rs}^2\right) \quad (10)$$

$$R_{rs}^1 / R_{rs}^2 = \max\left(\frac{Rrs(\lambda_1)}{Rrs(\lambda_4)}, \frac{Rrs(\lambda_2)}{Rrs(\lambda_4)}, \frac{Rrs(\lambda_3)}{Rrs(\lambda_4)}\right) \quad (11)$$

where λ_1 , λ_2 , λ_3 and λ_4 denote the wavelengths 443, 490, 510 and 555 nm, respectively. The coefficients were derived from a huge dataset (2804 station) with wide ranges of Chla concentrations (0.01 - 64 mg m^{-3}), while the majority of Chla concentrations were less than 10 mg m^{-3} [10].

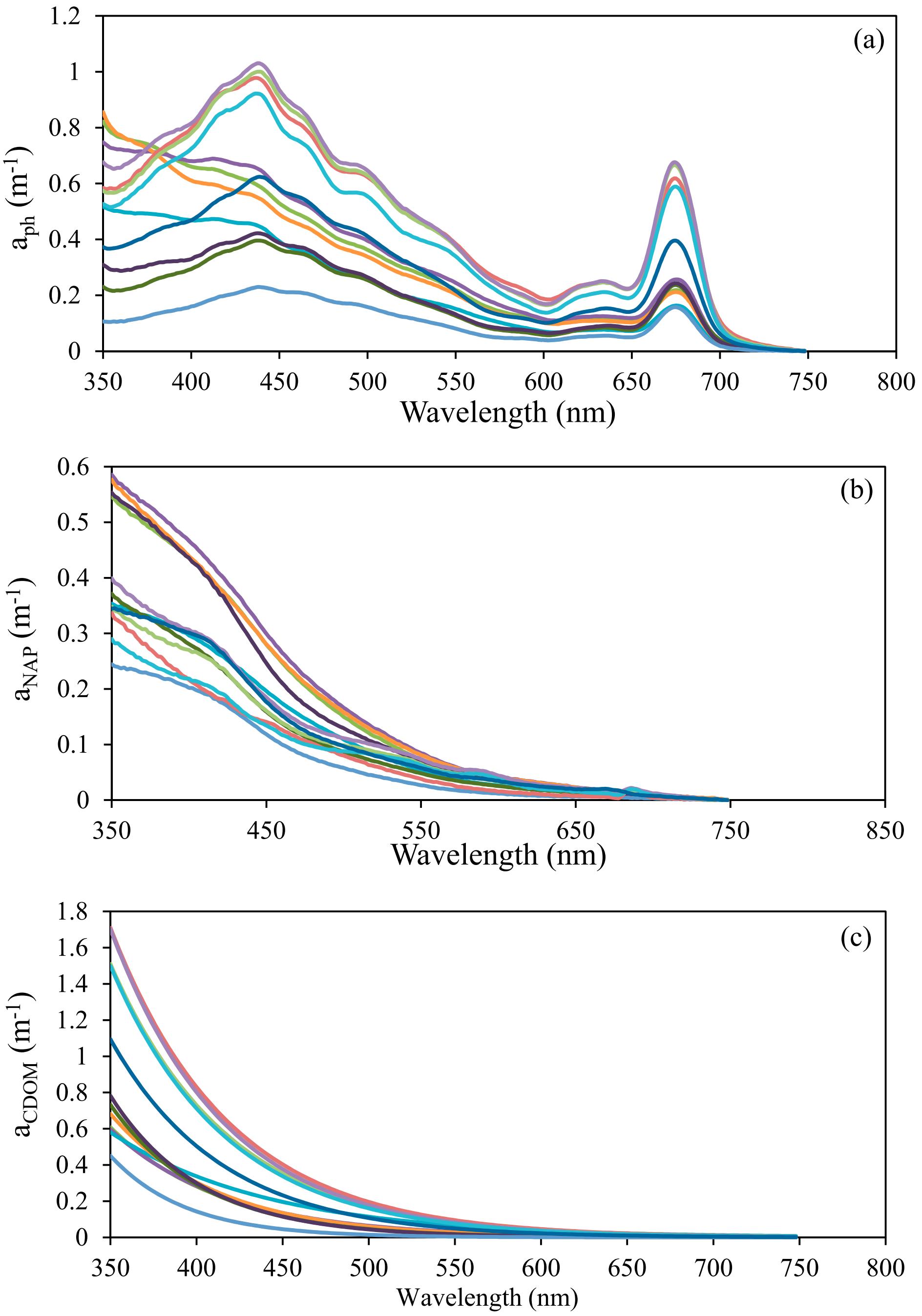

Fig. S1. Absorption spectra of (a) phytoplankton, a_{ph}, (b) non-algal particles, a_{NAP}, and (c) colored dissolved organic matter, a_{CDOM}, in Tokyo Bay (i.e., twelve stations with IOPs measurements).

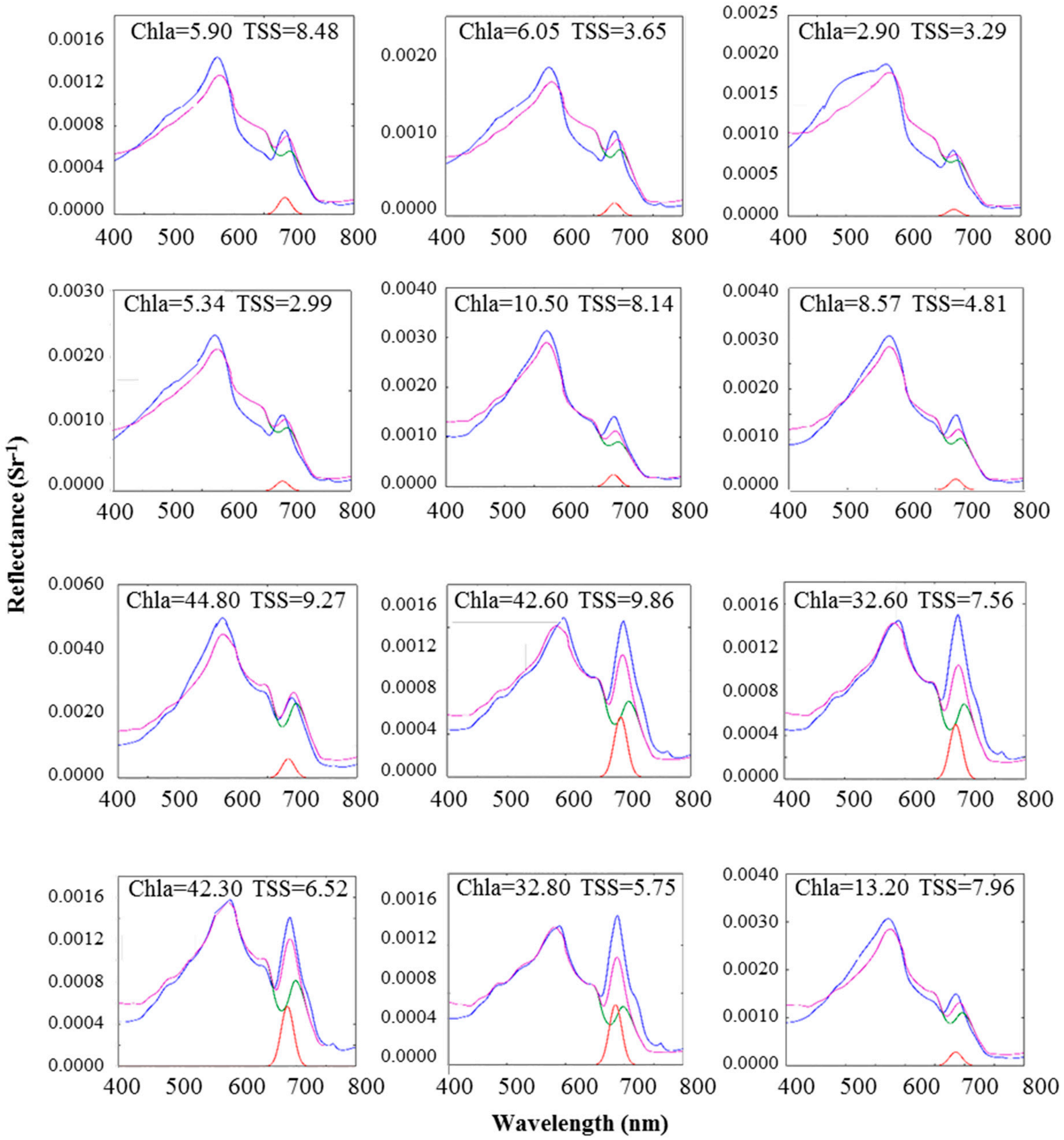

Fig. S2. Measured versus simulated remote sensing reflectance spectra at the twelve stations of Tokyo Bay with IOPs measurements. The blue and purple lines represent measured and simulated reflectance. The simulated reflectance spectrum is the summation of elastic reflectance (green line) and fluorescence reflectance (red line). Elastic reflectance refers to generating simulated reflectance by considering only total absorption and total backscattering without fluorescence reflectance. Chla and TSS refer to the concentrations of chlorophyll-a in mg m^{-3} and total suspended solids in g m^{-3}, respectively.

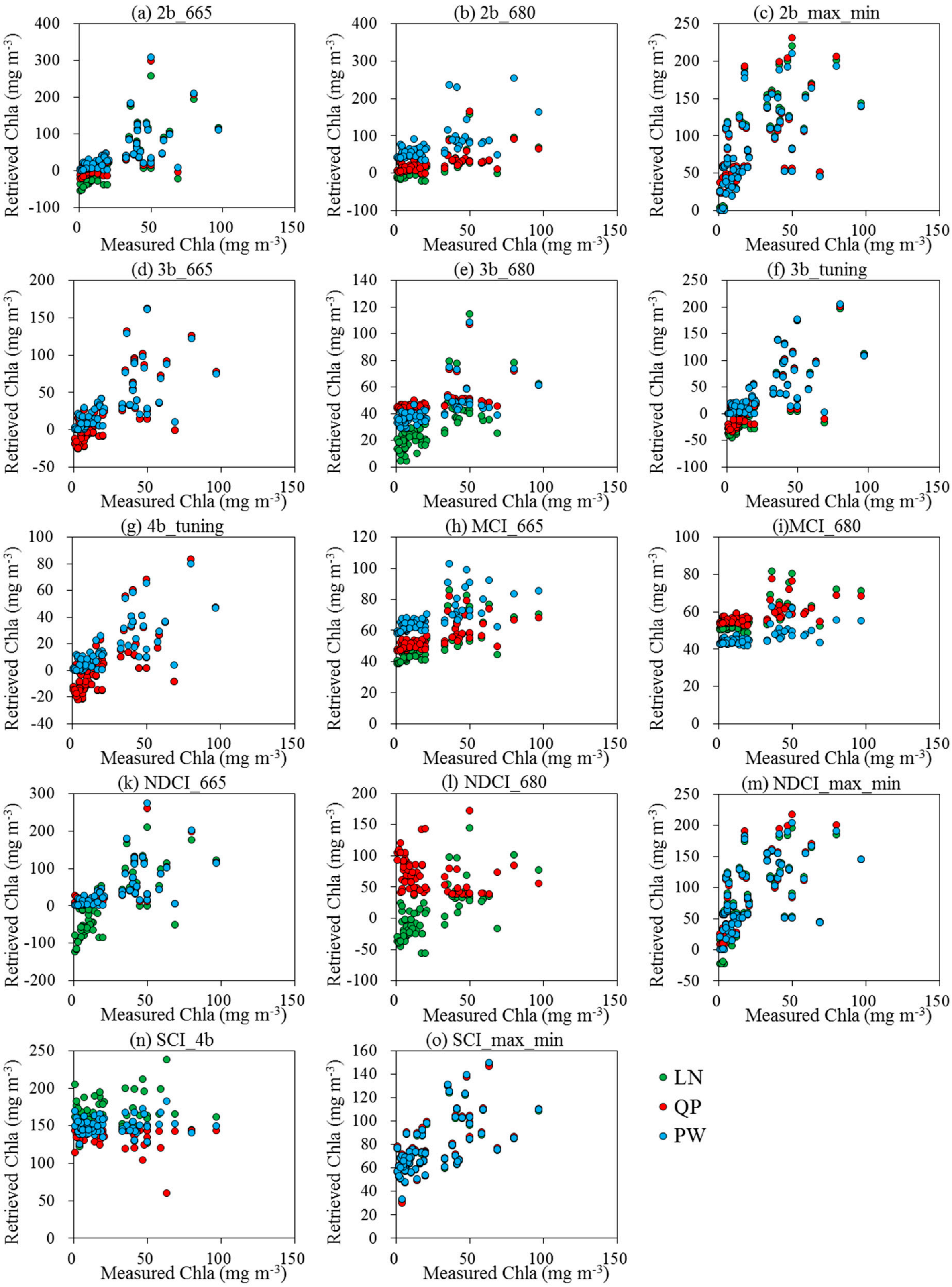

Fig. S3. Scatter plots between measured Chla in Tokyo Bay and retrieved Chla from models developed using simulated reflectance (Table 4 summarizes regression models).

Table S1

Accuracy assessment of simulated dataset's models (Table 4 summarizes regression models during calibration stage) using Tokyo Bay dataset.

Algorithms	R^2	RMSE	MARE
2b_665_LN	0.53	49.42	468.19
2b_665_QP	0.47	45.86	233.89
2b_665_PW	0.43	45.23	103.96
2b_680_LN	0.43	24.36	179.58
2b_680_QP	0.37	**21.26**	**86.53**
2b_680_PW	0.19	98.94	608.76
2b_max_min_LN	0.41	75.86	502.38
2b_max_min_QP	0.40	76.24	611.64
2b_max_min_PW	0.41	73.10	489.16
3b_665_LN	0.52	28.92	221.34
3b_665_QP	0.52	28.72	216.31
3b_665_PW	0.47	26.04	**86.08**
3b_680_LN	0.46	**17.56**	180.13
3b_680_QP	0.29	31.61	506.18
3b_680_PW	0.39	25.54	391.31
3b_tuning_LN	**0.56**	40.62	378.98
3b_tuning_QP	**0.56**	37.71	302.75
3b_tuning_PW	0.47	37.49	103.93
4b_tuning_LN	**0.57**	23.13	211.48
4b_tuning_QP	**0.57**	23.06	210.24
4b_tuning_PW	0.52	**17.14**	57.07
MCI_665_LN	0.53	31.73	484.96
MCI_665_QP	0.49	35.63	563.97
MCI_665_PW	0.50	47.79	727.00
MCI_680_LN	0.48	37.61	602.93
MCI_680_QP	0.44	39.29	635.36
MCI_680_PW	0.45	29.81	489.09
NDCI_665_LN	**0.54**	65.63	921.08
NDCI_665_QP	0.44	41.65	163.07
NDCI_665_PW	0.46	42.60	98.64
NDCI_680_LN	0.47	33.50	390.56
NDCI_680_QP	0.08	61.01	999.14
NDCI_680_PW	0.43	30.24	**94.42**
NDCI_max_min_LN	0.41	75.25	567.03
NDCI_max_min_QP	0.41	75.85	510.67
NDCI_max_min_PW	0.41	74.53	481.60
SCI_4b_LN	0.04	142.51	2094.01
SCI_4b_QP	0.07	118.46	1737.37
SCI_4b_PW	0.03	128.83	1908.33
SCI_max_min_LN	0.41	56.16	767.21
SCI_max_min_QP	0.41	57.38	780.95
SCI_max_min_PW	0.41	56.94	776.83

The highest three performing algorithms were highlighted in bold.

References

1. Gons, H. J. Optical Teledetection of Chlorophyll a in Turbid Inland Waters. *Environ. Sci. Technol.* **1999**, *33*, 1127–1132.

2. Mishra, S.; Mishra, D. R. Normalized difference chlorophyll index: A novel model for remote estimation of chlorophyll-a concentration in turbid productive waters. *Remote Sens. Environ.* **2012**, *117*, 394–406.

3. Dall'Olmo, G.; Gitelson, A. A.; Rundquist, D. C. Towards a unified approach for remote estimation of chlorophyll-a in both terrestrial vegetation and turbid productive waters. *Geophys. Res. Lett.* **2003**, *30*, 1938.

4. Zimba, P. V.; Gitelson, A. Remote estimation of chlorophyll concentration in hyper-eutrophic aquatic systems: Model tuning and accuracy optimization. *Aquaculture* **2006**, *256*, 272–286.

5. Le, C.; Li, Y.; Zha, Y.; Sun, D.; Huang, C.; Lu, H. A four-band semi-analytical model for estimating chlorophyll a in highly turbid lakes: The case of Taihu Lake, China. *Remote Sens. Environ.* **2009**, *113*, 1175–1182.

6. Gower, J.; King, S.; Borstad, G.; Brown, L. Detection of intense plankton blooms using the 709 nm band of the MERIS imaging spectrometer. *Int. J. Remote Sens.* **2005**, *26*, 2005–2012.

7. Gower, J.; King, S.; Goncalves, P. Global monitoring of plankton blooms using MERIS MCI. *Int. J. Remote Sens.* **2008**, *29*, 6209–6216.

8. Shen, F.; Zhou, Y.-X.; Li, D.-J.; Zhu, W.-J.; Suhyb Salama, M. Medium resolution imaging spectrometer (MERIS) estimation of chlorophyll- a concentration in the turbid sediment-laden waters of the Changjiang (Yangtze) Estuary. *Int. J. Remote Sens.* **2010**, *31*, 4635–4650.

9. O'Reilly, J. E.; Maritorena, S.; Mitchell, B. G.; Siegel, D. A.; Carder, K. L.; Garver, S. A.; Kahru, M.; McClain, C. Ocean color chlorophyll algorithms for SeaWiFS. *J. Geophys. Res.* **1998**, *103*, 24937.

10. O'Reilly, J. E.; Maritorena, S.; O'Brien, M. C.; Siegel, D. A.; Toole, D.; Menzies, D.; Smith, R. C.; Mueller, J. L.; Mitchell, B. G.; Kahru, M. SeaWiFS postlaunch calibration and validation analyses, part 3. *NASA Tech. Memo* **2000**, *2000–20689*, 3–8.
